# Frailty assessment in ANCA-associated vasculitis: current evidence and remaining uncertainties

**DOI:** 10.1093/rap/rkac078

**Published:** 2022-09-27

**Authors:** Choon Ying Wang, Henry H L Wu, Quinta Ashcroft, Lauren Floyd, Adam D Morris, Marwan Bukhari, Ajay P Dhaygude, Andrew C Nixon

**Affiliations:** Department of Renal Medicine, Lancashire Teaching Hospitals NHS Foundation Trust, Preston, UK; Department of Renal Medicine, Lancashire Teaching Hospitals NHS Foundation Trust, Preston, UK; Division of Cardiovascular Sciences, University of Manchester, Manchester, UK; Department of Renal Medicine, Lancashire Teaching Hospitals NHS Foundation Trust, Preston, UK; Department of Business Intelligence, Lancashire Teaching Hospitals NHS Foundation Trust, Preston, UK; Department of Renal Medicine, Lancashire Teaching Hospitals NHS Foundation Trust, Preston, UK; Division of Cardiovascular Sciences, University of Manchester, Manchester, UK; Department of Renal Medicine, Lancashire Teaching Hospitals NHS Foundation Trust, Preston, UK; Division of Cardiovascular Sciences, University of Manchester, Manchester, UK; Department of Rheumatology, University Hospitals of Morecambe Bay NHS Foundation Trust, Lancaster, UK; Department of Renal Medicine, Lancashire Teaching Hospitals NHS Foundation Trust, Preston, UK; Division of Cardiovascular Sciences, University of Manchester, Manchester, UK; Department of Renal Medicine, Lancashire Teaching Hospitals NHS Foundation Trust, Preston, UK

Rheumatology key message

- Further research efforts are needed to determine the most prognostically useful frailty assessment tool and the extent to which frailty assessment may guide treatment approaches for older ANCA-associated vasculitis populations.

Dear Editor, ANCA-associated vasculitis (AAV) is an uncommon autoimmune condition that typically affects older adults. From the age of 50 years onwards, the global incidence of AAV increases up to the age of 80 years [1]. The exact pathophysiology induced by ageing is not fully established, but immune dysregulation from cellular senescence is considered to play a significant role alongside other genetic and environmental factors [2]. Although the advancement of treatment options has significantly improved mortality outcomes in AAV, a high degree of morbidity as a consequence of this condition and its treatments remain. Frailty is an age-associated syndrome defined as a state of vulnerability to stressor events and thought to be the result of cumulative physiological decline [3]. The frailty syndrome is more commonly observed in older adults and those with chronic disease. A higher prevalence of AAV in older age groups suggested the need for frailty assessment to aid the prognostication and management of AAV in the older population.

Few studies have evaluated the relationship between frailty status and outcomes in AAV. A prospective observational study by McGovern *et al.* [4] published in *Rheumatology* analysed associations between baseline frailty status and mortality outcomes in AAV for 83 patients >65 years of age. Baseline frailty was determined prospectively by the clinical frailty scale (CFS) [5]. The CFS is a practical and easy-to-use subjective frailty assessment tool based on the deficit accumulation model and can be used in various clinical contexts [6]. Using the CFS, McGovern *et al.* [4] concluded that there were significant associations between baseline frailty status and mortality. The risk of death approximately doubled with each unit increase in CFS score (hazard ratio = 1.90, 95% CI = 1.03, 3.52).

We conducted a retrospective observational study aiming to investigate the associations between frailty status and health outcomes in patients with AAV using data collected from our centre over a 13-year period. Frailty status was assessed quantitatively using the hospital frailty risk score (HFRS) [7]. Derived from International Statistical Classification of Diseases and Related Health Problems, Tenth Revision (ICD-10) codes, the HFRS is a low-cost clinical assessment tool that could be applied systematically to identify frail people at risk of adverse outcomes using administrative hospital data when ICD-10 codes are in place [7]. It has demonstrated prognostic value in general older and advanced chronic kidney disease populations [7, 8]. Ethics application was not indicated in this study because patient identifiers were anonymized and only routinely collected data were used for analysis.

Thirty-four patients aged ≥75 years old and diagnosed with AAV in an inpatient or outpatient setting between 2008 and 2021 were included (Table 1). There were 15 female and 19 male patients, with mean age being 79.6 ± 3.8 years. The HFRS was calculated for each individual using hospital coding data from their initial AAV diagnosis consultation and up to two most recent hospitalizations before the AAV diagnosis, if any within the preceding 2 years [6]. Patients were categorized into three HFRS categories: low (<5), intermediate (5–15) and high (>15). There were 18, 13 and 3 patients in the low, intermediate and high HFRS groups, respectively. One or more of methylprednisolone, CYC and rituximab were given for patients across all three HFRS categories as remission induction therapy, with the exception of one patient in the low group and three in the intermediate group who did not receive any of these treatments. Ten (56%) patients in the low, five (38%) patients in the intermediate and all three (100%) patients in the high HFRS groups had one or more episodes of hospitalization after diagnosis of AAV. The median duration of hospitalization was 4.49 days in the low, 6.80 days in the intermediate and 28.8 days in the high HFRS groups. There was no statistically significant difference in the duration of hospitalization between HFRS groups (*P* = 0.185). Episodes of significant adverse events during hospitalization were identified in three (17%) patients in the low, one (8%) patient in the intermediate and one (33%) patient in the high HFRS group. Four out of five adverse event episodes were infection related, and the other was severe thrombocytopenia requiring intensive care admission. No patient in the high-scoring group received renal replacement therapy on presentation. Two and five patients in the low and intermediate groups received renal replacement therapy, respectively. Mortality outcomes were similar between the three groups. Four patients died within 1 year (low: two; intermediate: two; high: zero) and six patients by 2 years (low: three; intermediate: three; high: zero). The overall median duration of follow-up was 3.95 years. None of the patients included in analysis was lost to follow-up before the 1- and 2-year time points for which mortality was reported.

**Table 1.** Demographic and clinical data between the low, intermediate and high hospital frailty risk score groups

Parameter	Low HFRS (*n* = 18)	Intermediate HFRS (*n* = 13)	High HFRS (*n* = 3)	*P*-value^*^
	HFRS <5	HFRS 5–15	HFRS >15	
Age, mean (s.d.), years	78.6 (2.5)	81.1 (4.4)	81.3 (3.5)	0.249
Sex, female:male	8:10	6:7	1:2	0.921
HFRS, mean (s.d.)	2.2 (1.6)	8.1 (2.8)	17.7 (3.2)	<0.05
ELISA test distributions, *n* (%)				
MPO	9 (50)	11 (85)	3 (100)	0.058
PR3	5 (28)	2 (15)	0 (0)	0.458
Negative	4 (22)	0 (0)	0 (0)	0.133
Vasculitis manifestations, *n* (%)				
General	4 (22)	4 (31)	0 (0)	0.517
Cutaneous	3 (17)	2 (15)	0 (0)	0.749
Mucocutaneous/ophthalmic	0 (0)	0 (0)	0 (0)	–
ENT	2 (11)	1 (8)	0 (0)	0.820
Chest	3 (17)	4 (31)	0 (0)	0.413
Cardiovascular	1 (6)	1 (8)	0 (0)	0.875
Renal	12 (67)	9 (69)	2 (67)	0.988
Neurological	2 (11)	3 (23)	1 (33)	0.522
Distribution of induction immunosuppressive therapies administered, *n* (%)				
Methylprednisolone	10 (56)	10 (77)	3 (100)	0.207
CYC	10 (56)	8 (62)	2 (67)	0.907
Rituximab	6 (33)	1 (8)	2 (67)	0.071
Plasma exchange	4 (22)	1 (8)	0 (0)	0.399
Duration of hospitalization, median (IQR), days	4.49 (5.20)	6.80 (17.8)	28.8 (60.0)	0.185
Number of hospitalizations after diagnosis of ANCA-associated vasculitis, *n* (%)				
0	8 (44)	8 (62)	0 (0)	0.149
1 or 2	9 (50)	3 (23)	2 (67)	<0.05
>2	1 (6)	2 (15)	1 (33)	0.337
Significant adverse events, *n* (%)	3 (17)	1 (8)	1 (33)	0.498
Required RRT, *n* (%)	2 (11)	5 (38)	0 (0)	0.116
Mortality at 1-year follow-up, *n* (%)	2 (11)	2 (15)	0 (0)	0.624
Mortality at 2-year follow-up, *n* (%)	3 (17)	3 (23)	0 (0)	0.530
Duration of follow-up, median (IQR), years	3.95 (6.92)	4.95 (6.61)	1.49 (5.67)	0.426

* *P*-values are calculated using the chi-squared test for categorical variables and Kruskal–Wallis test for continuous variables.

HFRS: hospital frailty risk score; IQR: interquartile range; RRT: renal replacement therapy.

In summary, our findings did not illustrate the same associations between frailty status and mortality as concluded from the study by McGovern *et al.* [4]. Given the small sample size, it is not surprising that few statistically significant associations were found between HFRS scoring and mortality. There appear to be some potential associations, despite the lack of statistical significance from this underpowered study, between higher HFRS scoring and greater frequency/duration of hospitalizations, which will require confirmation with larger data samples. With a large number of different assessment tools available for the measurement of frailty status and a paucity of evidence specifically around frailty and AAV, more work is needed to determine the most prognostically useful frailty assessment tool in AAV populations and the extent to which frailty status might inform treatment strategies within this context. Anticipating the global growth of an ageing population living with varying degrees of multi-morbidity and frailty, an individualized frailty assessment and management approach is likely to be needed to optimize clinical outcomes for older patients.

## Data Availability

The data underlying this article will be shared on reasonable request to the corresponding author.
